# A methodological framework for the improved use of routine health system data to evaluate national malaria control programs: evidence from Zambia

**DOI:** 10.1186/s12963-014-0030-0

**Published:** 2014-11-19

**Authors:** Adam Bennett, Joshua Yukich, John M Miller, Penelope Vounatsou, Busiku Hamainza, Mercy M Ingwe, Hawela B Moonga, Mulakwo Kamuliwo, Joseph Keating, Thomas A Smith, Richard W Steketee, Thomas P Eisele

**Affiliations:** Malaria Elimination Initiative, Global Health Group, University of California, San Francisco, 550 16th St, San Francisco, CA 94143 USA; Center for Applied Malaria Research and Evaluation, Tulane University of Public Health and Tropical Medicine, 1440 Canal St., Suite 2200, New Orleans, LA 70112 USA; PATH Malaria Control and Evaluation Partnership in Africa (MACEPA), Lusaka, Zambia; Swiss Tropical and Public Health Institute, Socinstr. 57, 4051, Basel, Switzerland; University of Basel, Basel, Switzerland; National Malaria Control Centre, Ministry of Health, Lusaka, Zambia

**Keywords:** Malaria, Evaluation, Integrated Nested Laplace Approximation (INLA), Insecticide-treated nets, Health management information systems (HMIS)

## Abstract

**Background:**

Due to challenges in laboratory confirmation, reporting completeness, timeliness, and health access, routine incidence data from health management information systems (HMIS) have rarely been used for the rigorous evaluation of malaria control program scale-up in Africa.

**Methods:**

We used data from the Zambia HMIS for 2009–2011, a period of rapid diagnostic and reporting scale-up, to evaluate the association between insecticide-treated net (ITN) program intensity and district-level monthly confirmed outpatient malaria incidence using a dose–response national platform approach with district-time units as the unit of analysis. A Bayesian geostatistical model was employed to estimate longitudinal district-level ITN coverage from household survey and programmatic data, and a conditional autoregressive model (CAR) was used to impute missing HMIS data. The association between confirmed malaria case incidence and ITN program intensity was modeled while controlling for known confounding factors, including climate variability, reporting, testing, treatment-seeking, and access to health care, and additionally accounting for spatial and temporal autocorrelation.

**Results:**

An increase in district level ITN coverage of one ITN per household was associated with an estimated 27% reduction in confirmed case incidence overall (incidence rate ratio (IRR): 0 · 73, 95% Bayesian Credible Interval (BCI): 0 · 65–0 · 81), and a 41% reduction in areas of lower malaria burden.

**Conclusions:**

When improved through comprehensive parasitologically confirmed case reporting, HMIS data can become a valuable tool for evaluating malaria program scale-up. Using this approach we provide further evidence that increased ITN coverage is associated with decreased malaria morbidity and use of health services for malaria illness in Zambia. These methods and results are broadly relevant for malaria program evaluations currently ongoing in sub-Saharan Africa, especially as routine confirmed case data improve.

**Electronic supplementary material:**

The online version of this article (doi:10.1186/s12963-014-0030-0) contains supplementary material, which is available to authorized users.

## Introduction

As countries in sub-Saharan Africa (SSA) continue to scale up malaria control interventions with many moving toward elimination, rigorous evaluations are needed to ensure national programs are achieving desired impacts on malaria burden. While repeated national household surveys remain important for monitoring trends in population intervention coverage, their usefulness for assessing trends in the malaria burden will be limited in countries achieving low parasite prevalence where impractically large sample sizes are required to assess changes over time and across subnational areas [1]. As such, the use of routine health system data on malaria cases and deaths will become increasingly important for impact evaluation purposes. However, because of the known biases of routine malaria incidence data measured through health management information systems (HMIS) [2], these data have rarely been used to provide rigorous evidence of program effectiveness for decision-making in Africa [3].

Although time series HMIS data have been used for sophisticated climate modeling and early warning systems [4], to date most uses of HMIS data for program evaluation in Africa have been simple comparisons of pre- and post-intervention trends in rates of malaria case incidence and deaths [5]. Only in rare cases have such studies directly controlled for important confounding factors, including changing diagnostic confirmation practices, access and use of health services, HMIS completeness, and rainfall and temperature, all of which likely lead to biased findings of program effectiveness [2,6].

In addition, a particular issue with evaluating the impact of national malaria control programs is that they normally attempt to cover all at-risk populations with interventions, which precludes the availability of a contemporaneous control group. This challenge of evaluating full-coverage programs is by no means unique to malaria or public health. As a possible solution to this challenge, Victora and colleagues (2011) proposed an evolution in evaluation design for large-scale health programs that uses the district as the unit of analysis to test for a dose–response relationship between program inputs (or coverage) and health outcomes, referred to by the authors as a national platform analysis [7]. Graves and colleagues (2008) previously used such an approach in their evaluation of vector control scale-up in Eritrea on the outcome of HMIS-derived malaria case incidence, while accounting for climate variability [8]. However, while their study is a significant advancement over simple analysis of HMIS trends over time, they did not account for malaria diagnosis practices, health services access, treatment-seeking, and spatial and other unobserved correlations in the data.

Zambia has successfully scaled up insecticide-treated mosquito nets (ITNs) since 2005, with 64% of households owning at least one as of 2010 [9]. Since 2009, Zambia has achieved national-level access to rapid diagnostic tests (RDTs) and has invested substantial resources at improving HMIS malaria data collection and reporting. As a result, Zambia provides an example where prevalence was historically high, but effective control has achieved an environment of intervention-suppressed transmission, and confirmed case data from HMIS are increasingly available in addition to survey prevalence data to measure trends in the malaria burden. However, because of the recent scale-up of RDTs and improved health access, use of HMIS case incidence to evaluate malaria program performance must account for improving diagnostic confirmation, HMIS reporting, and access to health services, or results could erroneously suggest the malaria burden is getting worse as malaria control interventions are scaled up.

Here we present results from a district-level evaluation design that was used to assess the dose–response relationship between ITN program intensity and HMIS-derived confirmed malaria case incidence in Zambia between 2009 and 2011. In doing so, we present a novel framework for rigorously evaluating full-coverage malaria programs, as well as child survival programs in general, that rely on imperfect HMIS data, by controlling for variability in diagnostic procedures, completeness of reporting, access and demand for health services, and climate, while accounting for the inherent correlation of these types of data across time and space.

## Methods

### Study site

Zambia has been scaling up coverage of long-lasting ITNs (LLINs), indoor residual spraying (IRS), prompt and effective treatment with artemisinin-combination therapies (ACTs), and diagnosis at point-of-care with RDTs since 2006 [10]. The proportion of households with at least one ITN increased from 38% in 2006 to 62% in 2008 and 64% in 2010; the proportion of households receiving IRS in the past 12 months increased from 10% in 2006 to 15% in 2008 and 23% in 2010; [9] RDT scale-up has allowed for confirmed diagnosis at the majority of facilities nationally since 2009 [11], and the HMIS reporting system was overhauled in 2008, which has greatly strengthened routine reporting. Zambia is divided administratively into 74 districts within 10 provinces (72 and nine for the current analysis due to an administrative separation in 2011); as of 2011, a total of 1,695 public facilities (96 hospitals, 1,352 health centers/clinics, and 247 health posts) and 35 non-governmental clinics reported into the HMIS on a monthly basis. Reporting for malaria includes clinical and confirmed outpatient cases, inpatient cases, deaths, laboratory testing, and commodity use.

### Study design and participants

A dose–response ecological analysis was conducted with district-months as the unit of analysis to evaluate the association between ITN program intensity and outpatient malaria case incidence. Data from the Zambia HMIS on all monthly reported confirmed and clinical (unconfirmed) outpatient malaria cases from 2009–2011 were included. Data before 2009 were excluded as cases up to this point were reported only on a quarterly basis, a large proportion of facilities did not report, and parasitological confirmation was not widespread or reported. Strengthening the Reporting of Observational studies in Epidemiology (STROBE) guidelines were followed for the reporting of methods and results [12].

### Primary outcomes

**Figure 1:**
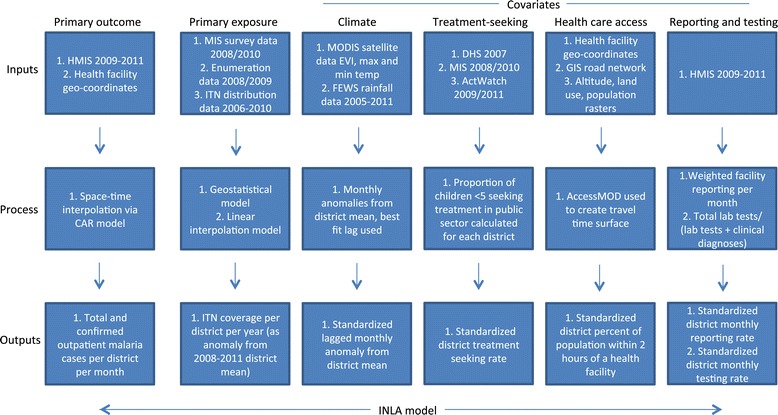
**Conceptual diagram of model inputs, processes, and outputs.**

A conceptual diagram of steps taken to create all variables for analysis is provided in Figure 1, and detailed description of data preparation is provided in Additional file 1. The primary outcomes included monthly confirmed and total (confirmed + unconfirmed) outpatient malaria cases aggregated at the district level. Before aggregating to the district level, we imputed all missing facility-level monthly outpatient malaria values based upon the spatial location of the facility and the month in which it occurred using Bayesian conditional autoregressive models.

### Measures of primary exposure variables

The primary exposure variable for this analysis was ITN coverage measured as ITNs per household at the district level per year. Bayesian geostatistical models were first used to produce estimates of ITN per person ratios from National Malaria Indicator Surveys (MIS) and IRS program enumeration efforts in 2008 and 2010 [9,13], and population-adjusted values were calculated per district (see Additional file 1: Figures S1 and S2). Bayesian generalized linear models were then used to predict values of ITN per person ratios for districts and years without survey data from annual district ITN distribution data from the National Malaria Control Center (NMCC) (Additional file 1: Figure S3). The resultant district-level ITN per person ratio was multiplied by the average household size of each district in order to represent population coverage as a more programmatically useful value, the number of ITNs per household. In final regression models, we included this number of ITNs per household variable as an anomaly from the four-year mean for each district to control for systematic spatial effects and potentially endogenous relationships due to programmatic targeting decisions.

Program data on the annual numbers of structures sprayed with IRS per district were compiled to investigate independent effects of spraying and as a control variable. In preliminary models, we found IRS terms to be non-significant and positively associated with incidence, which likely reflects a high degree of endogeneity given that the IRS program initially targeted peri-urban areas and were scaled up in higher burden areas. As we were unable to identify an effective instrumental variable for IRS, we only retained IRS as a control variable—calculated as an anomaly from the four-year district mean of the number of structures sprayed in the previous year—and did not attempt to interpret independent associations with incidence.

### Measures of contextual and potential confounding factors

To control for climate variability over the study period, monthly climatic data were compiled from publicly available sources at the district level. Monthly mean rainfall data were obtained from the Famine Early Warning System African Data Dissemination Service [14] from 2005 through 2011. Monthly mean maximum and minimum temperature and the enhanced vegetation index (EVI) were obtained from MODIS satellite data for the same period [15]. Based upon exploratory analyses, EVI values were categorized as <0 · 2, 0 · 2–0 · 3, 0 · 3–0 · 4, and >0 · 4, where higher values represent greater vegetation health. Anomalies were calculated for rainfall and temperature data as the difference from each district-month value and the district mean. Rainfall and temperature anomaly values were then standardized by subtracting the respective overall mean and dividing by the standard deviation. For inclusion in regression models, various month lag terms were assessed according to previously documented lagged relationships between climate variables and clinical incidence [16].

To estimate physical access to health care, the open source module AccessMOD 3.0 [17] was used to create smoothed raster estimates of travel time to health facilities by district. This estimate of facility access has been shown to correlate well with treatment-seeking for fevers from MIS data [18]. Based upon these estimates and district population rasters, we calculated the percent of each district population within two hours of a public health facility (Additional file 1: Figure S4). These values were standardized for inclusion in final regression models by subtracting the overall mean and dividing by the standard deviation.

Data from the 2006, 2008, and 2010 MIS, 2007 Demographic and Health Survey [19], and 2009 and 2011 ACTWatch household surveys [20] were compiled to estimate rates of treatment-seeking for fever per district. For each district the proportion of caregivers from all six surveys who sought treatment at a public health facility for a child <5 with fever was calculated (Additional file 1: Figure S5). We examined simple kriging methods but found no difference with these cross-survey district summaries. Similar to rainfall, temperature, and health care access, district mean treatment-seeking rates were standardized to one standard deviation.

To evaluate reporting rates over time we created an index of the number of facilities reporting per district per month as a proportion of the total number of facilities per district, weighted by facility size (determined by mean monthly malaria outpatient diagnoses over the study period). We created a similar index for testing per district-month calculated as the total number of parasitological tests (slide or RDT) reported per health facility per month divided by the sum of the total number of tests and the total number of clinical (non-confirmed) malaria cases. In cases where a confirmed case count was reported but no parasitological testing value reported (roughly 33% of all testing values), we replaced the missing testing value with the number of confirmed cases.

### Statistical analysis

For descriptive analyses, confirmed case data were standardized per 1,000 population and summarized as the annual parasite index (API), which is commonly used outside Africa [21] but only rarely used in Africa due to low case confirmation rates. Mid-year district-level population estimates were available from the 2010 housing and population census and projected for 2009 and 2011 based upon annual rates of change. We compared several Poisson and negative binomial regression models to test the association between ITN coverage per district and the primary outcomes of total and confirmed malaria outpatient cases. In all models, we used the fully imputed cases and included the log of the total district population as a measure of exposure in order to create population-standardized incidence rates. Exploratory and residual analysis revealed potential interactions by region between primary outcome and explanatory variables. In model construction we therefore assessed the inclusion of interactions between ITN coverage and transmission, as measured by mean *P. falciparum* parasite rate (*Pf*PR_2–10_) (Malaria Atlas Project) categories (<10% vs. >10% and <25% vs. >25%), as well as between ITN coverage and high-burden/low-burden province, where high-burden provinces were those with the highest confirmed case incidence over the entire period (Luapula, Copperbelt, and Eastern provinces as defined in 2011) (Additional file 1: Figure S6). Models were fit in a Bayesian framework and computed using Integrated Nested Laplace Approximation (INLA) in R to account for unmeasured temporal and spatial correlation [22,23]. Model fit was compared using the deviance information criterion (DIC) [24], where models with the lowest DIC were chosen for final interpretation. Where uncertainty from the INLA model did not include zero, coefficients were considered significantly different than zero. As a further check on model specification, we compared the results of models fit by INLA with models fit in a frequentist framework and obtained similar coefficient estimates.

## Results

**Figure 2:**
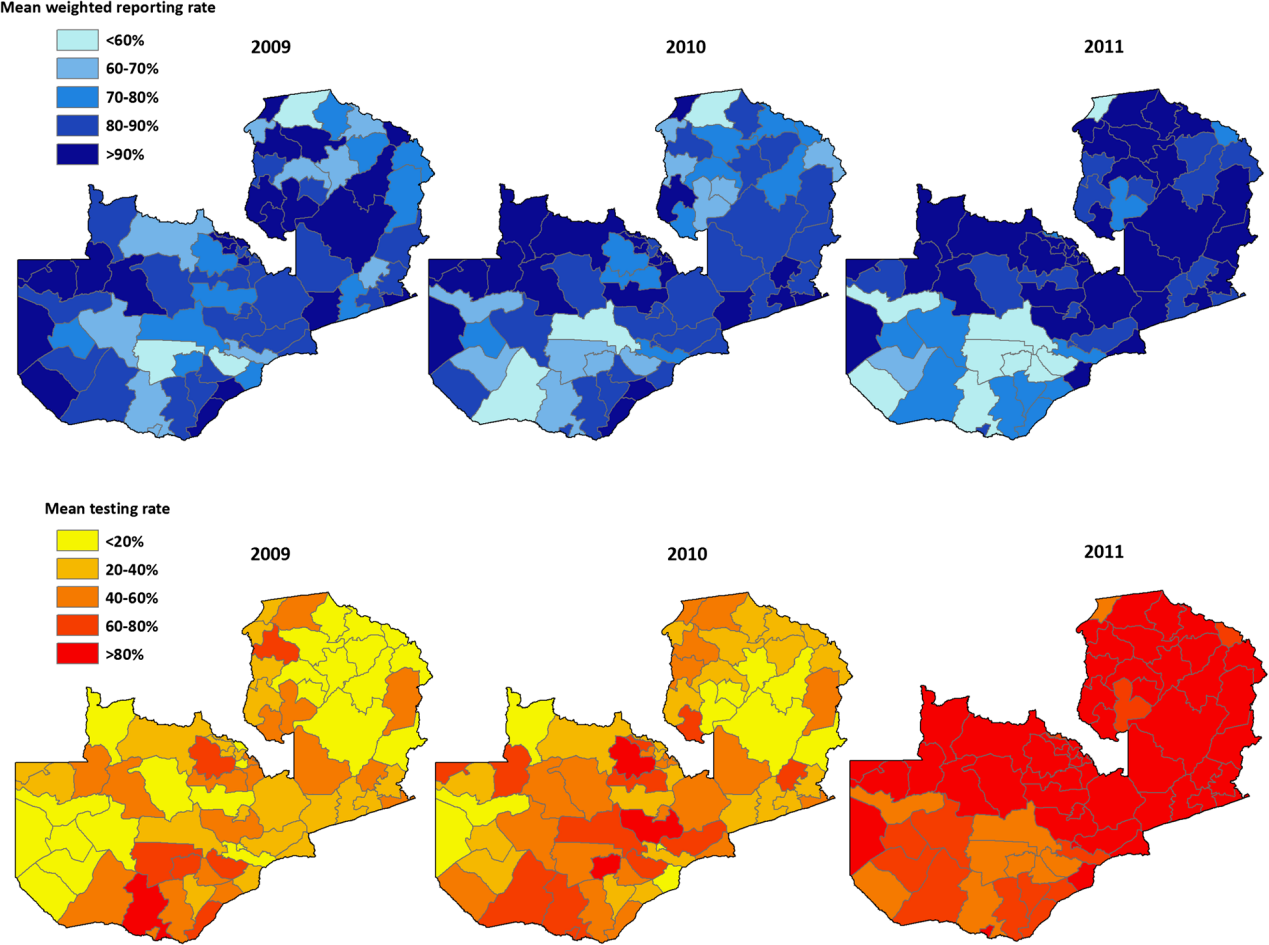
**Mean weighted reporting rate and mean testing rate (defined as the number of tests reported divided by the sum of tests reported and clinical cases) by district for 2009, 2010, and 2011, Zambia.**

The 2009–2011 HMIS data set included 1,693 facilities that reported at least one malaria observation, of which we were able to geo-reference with global positioning systems (GPS) 1,387 (82%); the remaining 306 (18%) were matched to district. Of the 60,948 maximum possible facility-month observations, there were 48,166 (79.0%) non-missing values available for total malaria cases and 38,588 (63.3%) non-missing values for confirmed cases alone; the remaining 21.0% of total cases and 36.7% of confirmed case values were imputed. The percent of expected reports of values per year was consistent over the study period among health centers (2009: 84 · 7%, 2010: 85 · 1%, 2011: 84 · 2%) and hospitals (2009: 65 · 1%, 2010: 62 · 9%, 2011: 63 · 3%) but increased among health posts (2009: 54 · 4%, 2010: 67 · 1%, 2011: 77 · 4%). The mean weighted district-level reporting rate increased slightly from 81 · 1% in 2009 to 84 · 6% in 2011 but fell somewhat in some districts at the end of 2010 and 2011 (Figure 2). Consistent with the rapid scaling-up of testing and reporting with RDTs in clinics across Zambia over this period, the mean testing rate (defined as the number of tests reported divided by the sum of tests reported and clinical cases) increased dramatically over this period, from 33 · 0% in 2009 to 43 · 2% in 2010 and 67 · 6% in 2011. This increase in uptake and reporting of testing was largely consistent across districts.

**Figure 3:**
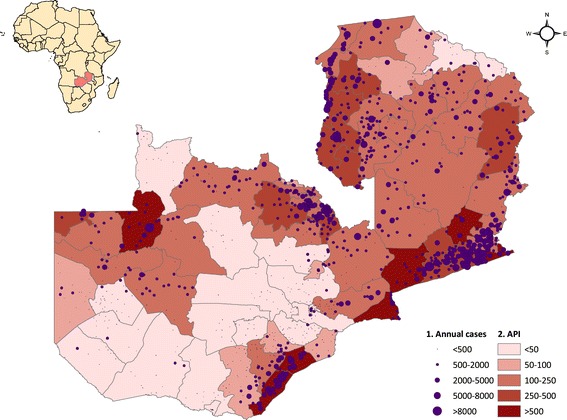
**(1) Annual confirmed outpatient cases by facility and (2) annual parasite index (API) by district after imputing missing facility-month values, three-year average 2009–2011, Zambia.**

**Figure 4:**
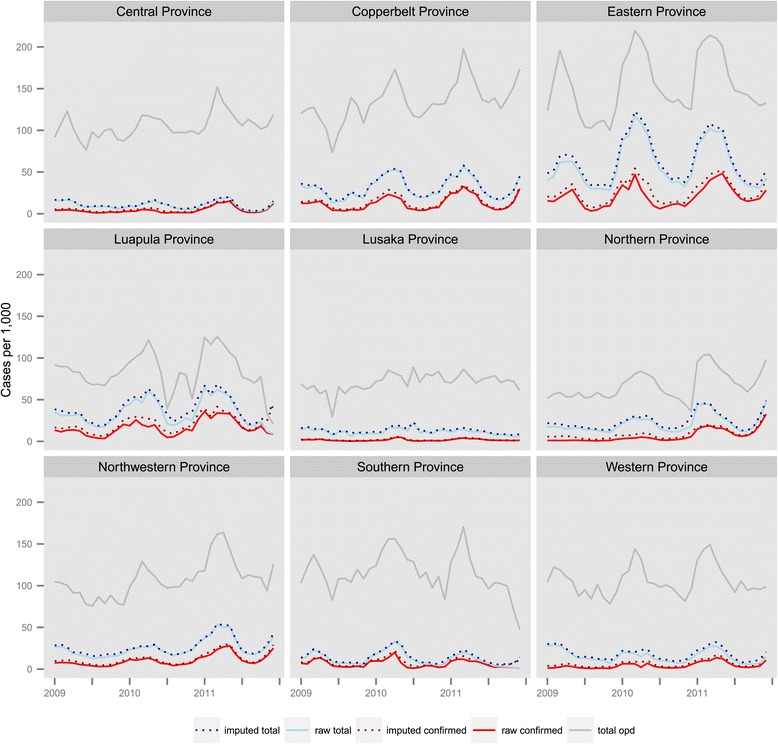
**Total (clinical + confirmed) and confirmed outpatient malaria case incidence, and total all-cause outpatient incidence, per 1,000 population by province and month, 2009–2011, Zambia.**

Total outpatient malaria cases (clinical and confirmed) reported through the HMIS were concentrated in districts on the south-eastern border with Zimbabwe, Mozambique, and Malawi, as well as in Luapula, Northern, Copperbelt, and portions of Northwestern Provinces (Figure 3). Coinciding with the progressive roll out of the new HMIS reporting system, total reported outpatient malaria cases increased from 3 · 0 million in 2009 (242 · 2 per 1,000 population) to 4.1 million in 2010 (322 · 8 per 1,000 population) and 4 · 3 million in 2011 (327 · 5 per 1,000 population). After imputing missing monthly facility case values, there were an estimated 3 · 4 million outpatient malaria cases in 2009 (277 · 4 per 1,000 population), 4 · 6 million in 2010 (360 · 0 per 1,000 population), and 4 · 7 million in 2011 (361 · 9 per 1,000 population). Coinciding with the scale-up of diagnostic testing for malaria confirmation, confirmed outpatient malaria cases reported through the HMIS also increased, from 871,193 cases in 2009 (API = 70 · 8 per 1,000 population) to 1 · 2 million in 2010 (API = 97 · 4) and 2 · 1 million in 2011 (API = 163 · 0). After imputing missing monthly facility case values, there were 1.2 million confirmed malaria cases in 2009 (API = 99 · 8), 1 · 7 million in 2010 (API = 135 · 9), and 2 · 5 million in 2011 (API = 194 · 8). Although reported confirmed case incidence increased in most provinces from 2010 to 2011, total case incidence decreased in Southern Province and slightly in Eastern Province where incidence is highest (Figure 4).

District-level ITN coverage, as measured by the number of ITNs per household, increased from 1 · 25 in 2009 to 1 · 34 in 2010 but fell slightly in 2011 to 1 · 28. District-level ITN coverage and confirmed malaria case incidence showed great variability, with some districts experiencing drops in ITN coverage associated with an increase in confirmed case incidence and others experiencing substantial increases in ITN coverage and stable or decreasing incidence (Additional file 1: Figure S7).

After controlling for district reporting and testing rates, the percent of the population within two hours of a health facility, mean treatment-seeking, IRS, rainfall, minimum and maximum temperature, vegetation, calendar month and year, and spatial and temporal autocorrelation, overall the number of ITNs per household was significantly associated with lower confirmed case incidence iIncidence rate ratio (IRR) = 0 · 73, 95% Bayesian Credible Interval (BCI): 0 · 65–0 · 81) (Table 1 and Additional file 1: Table S1). In low-burden regions, the number of ITNs per household was strongly associated with lower confirmed case incidence (IRR = 0 · 59, 95% BCI: 0 · 51–0 · 68); there was no evidence of this association in high-burden provinces (IRR = 0 · 94, 95% BCI: 0 · 79–1 · 10). Similarly, the number of ITNs per household was associated with lower total malaria case incidence in the overall model (IRR = 0 · 69, 95% BCI: 0 · 62–0 · 76), as well as in low-burden provinces (IRR = 0 · 53, 95% BCI: 0 · 46–0 · 62), but not in high-burden provinces (IRR = 0 · 93, 95% BCI: 0 · 81–1 · 10).

**Table 1:** Results of space-time negative binomial models fit using INLA, for overall models (1) and models including interaction by region (2), Zambia*

**Characteristic**	**Adjusted model coefficients (IRR, 2 · 5%-97 · 5%)**			
	**Confirmed cases (1)**	**Confirmed cases (2)**	**Total cases (1)**	**Total cases (2)**
**ITNs per HH (overall)**	0 · 73 (0 · 65–0 · 81)		0 · 69 (0 · 62–0 · 76)	
**ITNs per HH in low burden**		0 · 59 (0 · 51–0 · 68)		0 · 53 (0 · 46–0 · 62)
**ITNs per HH in high burden**		0 · 94 (0 · 79–1 · 10)		0 · 93 (0 · 79–1 · 10)
**Reporting rate** ^**Ŧ**^	0 · 99 (0 · 97–1 · 00)	0 · 98 (0 · 97–1 · 00)	1 · 07 (1 · 05–1 · 09)	1 · 07 (1 · 05–1 · 09)
**Testing rate** ^**Ŧ**^	1 · 22 (1 · 19–1 · 25)	1 · 22 (1 · 19–1 · 25)	0 · 87 (0 · 85–0 · 89)	0 · 87 (0 · 85–0 · 89)
**Percent of population within 2 hrs of public health facility** ^**Ŧ**^	0 · 77 (0 · 60–0 · 98)	0 · 77 (0 · 61–0 · 97)	0 · 85 (0 · 67–1 · 07)	0 · 84 (0 · 67–1 · 07)
**Treatment-seeking rate** ^**Ŧ**^	1 · 03 (0 · 84–1 · 27)	1 · 03 (0 · 85–1 · 26)	1 · 02 (0 · 83–1 · 26)	1 · 03 (0 · 84–1 · 27)
**High-burden province (ref: low)**	2 · 39 (1 · 27–4 · 52)	2 · 39 (1 · 31–4 · 36)	2 · 08 (1 · 13–3 · 84)	2 · 09 (1 · 13–3 · 85)
**IRS** ^**Ŧ**^	1 · 04 (1 · 02–1 · 06)	1 · 05 (1 · 03–1 · 06)	1 · 01 (0 · 99–1 · 02)	1 · 01 (0 · 99–1 · 03)
**RFE (2–3 months lag)** ^**Ŧ**^	0 · 99 (0 · 97–1 · 01)	0 · 99 (0 · 98–1 · 01)	0 · 99 (0 · 97–1 · 01)	0 · 99 (0 · 97–1 · 01)
**Max temp (2 mo. lag)** ^**Ŧ**^	1 · 02 (1 · 00–1 · 04)	1 · 02 (1 · 00–1 · 04)	1 · 03 (1 · 01–1 · 05)	1 · 03 (1 · 01–1 · 05)
**Min temp (2 mo. lag)** ^**Ŧ**^	1 · 01 (0 · 99–1 · 03)	1 · 01 (0 · 99–1 · 03)	1 · 00 (0 · 98–1 · 02)	1 · 00 (0 · 99–1 · 02)
**EVI**				
<0 · 2 (ref)				
0 · 2–0 · 3	1 · 15 (1 · 08–1 · 21)	1 · 15 (1 · 08–1 · 21)	1 · 12 (1 · 06–1 · 19)	1 · 12 (1 · 06–1 · 19)
0 · 3–0 · 4	1 · 29 (1 · 18–1 · 40)	1 · 29 (1 · 18–1 · 41)	1 · 32 (1 · 21–1 · 44)	1 · 33 (1 · 22–1 · 45)
>0 · 4	1 · 35 (1 · 22–1 · 51)	1 · 36 (1 · 22–1 · 51)	1 · 37 (1 · 23–1 · 52)	1 · 38 (1 · 24–1 · 53)
**Year**				
2009 (ref)				
2010	1 · 38 (0 · 88–2 · 16)	1 · 41 (0 · 90–2 · 21)	1 · 23 (0 · 80–1 · 90)	1 · 27 (0 · 83–1 · 95)
2011	1 · 89 (0 · 82–4 · 37)	1 · 90 (0 · 83–4 · 36)	1 · 46 (0 · 66–3 · 25)	1 · 47 (0 · 66–3 · 27)
**DIC**	38241 · 0	38225 · 8	42901 · 9	42878 · 1
**N**	2592	2592	2592	2592

*models include calendar month dummy covariates ^Ŧ^covariates are standardized so that a one-unit change represents one standard deviation INLA = Integrated Nested Laplace Approximation; IRR = incidence rate ratio; ITNs = insecticide-treated nets; HH = household; IRS = indoor residual spraying; RFE = rainfall estimate; EVI = enhanced vegetation index; DIC = deviance information criterion.

The standardized testing rate was positively associated with confirmed malaria case incidence (IRR = 1 · 22, 95% BCI: 1 · 19–1 · 25) but was negatively associated with total malaria case incidence (IRR = 0 · 87, 95% BCI: 0 · 85–0 · 89). The standardized reporting rate was positively associated with total malaria case incidence (IRR = 1 · 07, 95% BCI: 1 · 05–1 · 09) but not with confirmed case incidence (IRR = 0 · 98, 95% BCI: 0 · 97–1 · 00). One-month lagged EVI was positively associated with confirmed case incidence (IRR = 1 · 36 comparing the highest and lowest EVI categories, 95% BCI: 1 · 22–1 · 51). The standardized percent of the population within two hours of a health facility was inversely associated with confirmed case incidence (IRR = 0 · 77, 95% BCI: 0 · 61–0 · 97), which likely reflects the proportion of the population in urban areas in each district.

## Discussion

In this study, we used a national platform evaluation design to assess the dose–response relationship between district-level ITN program intensity and HMIS-derived confirmed malaria case incidence in Zambia between 2009 and 2011. After accounting for variability in diagnostic procedures, completeness of reporting, and access and demand for health services, we show that increased district-level ITN coverage as measured by the number of ITNs per household is associated with lower confirmed case incidence. Specifically, we found that an additional ITN per household was associated with a 27% reduction in district-level monthly confirmed case incidence overall and a 41% reduction in provinces with lower annual burden. This finding is largely consistent with field trials and corresponds to an average of over 300,000 fewer confirmed outpatient malaria cases per year with each additional ITN per household [25].

This study illustrates a robust framework for mitigating many of the known biases of routine data on malaria incidence by controlling for important confounding factors, which is a prerequisite to achieving the high internal validity required for rigorous program evaluations. Prior analyses of similar routine data have often failed to control for important confounders, and many have reported on presumed or clinically-diagnosed malaria cases. For example, Otten and colleagues (2009) found large reductions in the number of cases in Rwanda and Ethiopia and attributed these changes to the scale-up of malaria prevention interventions [26]. Similarly, Chanda and colleagues (2012) analyzed annual HMIS data summaries from a sample of districts in Zambia for 2007 and 2008 and concluded that ITNs and IRS were associated with declines in suspected malaria case incidence and deaths [5]. However, neither of these studies adequately controlled for several important confounding factors known to influence health facility incidence, including variations over time in climate, diagnostic practices, access to health services, treatment-seeking behavior, and reporting completeness [2]. Bhattarai and colleagues (2007) found decreases in health facility cases following LLIN and ACT scale-up in Zanzibar, but while climate was considered descriptively, it was not explicitly modeled, and the authors did not consider differences in treatment-seeking or health care access [27]. The district level (“sub-zobas” in Eritrea) analysis conducted by Graves and colleagues (2008) revealed an association between the number of ITNs distributed, IRS spraying, and clinical case incidence, but while their analysis controlled for climate factors, they did not include information on parasitological case confirmation, reporting, or health facility access and treatment-seeking behavior [8]. Finally, none of these studies accounted for the inherent correlated nature of malaria case data across spatial units, which can result in erroneous findings of statistical significance if not accounted for, and only the Graves study accounted for temporal autocorrelation.

This study incorporated several recent methodological advances in spatial and spatio-temporal modeling that allow for the inclusion of complex correlation structures, as well as spatially continuous intervention and environmental information [28,29]. Similar modeling strategies have increasingly been used to evaluate temporal and spatial trends in disease, seasonality, climate, and other factors but not for evaluations of program impact [30]. While the evaluation framework and accompanying statistical analyses used in this study are complex, we argue that without such methods in place to account for potential biases in routine HMIS data, such data cannot be used for rigorous program evaluations to achieve meaningful and robust results for program decision-making. This is significant as HMIS data become increasing available, parasite prevalence falls in areas with high control coverage, RDTs are scaled up to allow for increased case confirmation, and as programs require better real-time data to monitor trends in confirmed cases and deaths.

Our finding of a significant interaction between the number of ITNs per household and low versus high incidence regions in models predicting both confirmed and all malaria outpatient cases was unexpected; while potentially related to transmission, we did not find significant interactions between district ITN coverage and endemicity categories as defined by mean *Pf*PR_2–10_. Mathematical models and some limited empirical evidence have suggested that the effect of increasing ITN coverage on prevalence may be greater or more rapid in areas of lower baseline transmission [25,31-33], but there is less evidence to suggest a similar relationship with clinical case incidence. It is possible that regional factors such as population movement between neighboring countries or insecticide resistance are involved, as these provinces border high burden areas in Malawi, Mozambique, and the Democratic Republic of the Congo where resistance is a known problem reducing operational effectiveness [34], and reductions in the malaria burden over a decade of scale-up have been extremely limited [35]. It is also possible that our testing and reporting rates do not fully correct for biases in diagnostic reporting practices in these high-burden areas.

The increase in both confirmed and total malaria outpatient cases over the period of study is notable yet largely explained by the rapid increase in RDT testing over this period and a simultaneous increase in reporting of confirmed cases as a new HMIS reporting system was adopted. Inter-annual climate patterns may explain some of the increase between 2009 and 2010, as 2010 was noted as a high transmission year in several countries in the region [36], but we found limited evidence for this effect in our models. User fee changes adopted in 2006 may have influenced health facility utilization rates broadly, but the bulk of these effects would likely have well predated our study.

There were several important limitations to our approach that should be considered. First, our evaluation was limited by the short time frame of confirmed case data available for analysis, as well as potentially biased by the increase in reporting and testing over this period as facilities adapted to the new reporting system. While we attempted to control for the increase in confirmed case testing in multivariable models, our testing rate may be an imperfect indicator of the true testing rate, as reporting of testing likely improved contemporaneously with RDT scale-up, laboratory testing values were not consistently reported, and detailed RDT stock-out data were not available. However, any remaining bias would most likely bring the estimated effect of ITNs on confirmed case incidence toward the null hypothesis of no effect.

Second, potentially endogenous relationships existed between our primary outcomes and explanatory variables of interest due to programmatic choices targeting high-burden or easily accessible areas. In some instances, such as the use of calendar month in the evaluation of IRS effectiveness by Over and colleagues [37], instrumental variables may be available to infer causal relationships when endogeneity exists. However, as no instrumental variables uncorrelated with primary outcomes were available in our data, we were not able to perform two-stage regression or similar standard econometric approaches to isolate uncorrelated effects. Rather, we controlled for systematic spatial targeting of intervention effort through the use of anomalies in program coverage. This approach was effective for ITN coverage, as the goal is for universal coverage, and therefore targeting has been limited. However, the highly targeted nature and relatively lower coverage of the IRS program during this period, combined with the lack of confirmed case data preceding IRS scale-up, precluded our ability to make similar effective adjustments for IRS. Future use of these data will likely prove more robust for evaluating IRS efforts as more areas are included and there is greater heterogeneity within districts over time. Additionally, we incorporated only annual ITN per household data, which may not accurately depict monthly changes in coverage. There is need for programs to more closely track monthly ITN coverage data in order to make more temporally refined assessments of intervention effectiveness.

Finally, we were not able to incorporate ACT data in this analysis as these data were not available sub-nationally. Drug stock-outs could have influenced incidence rates, but there was no evidence to suggest systemic changes in ACT availability over this period.

## Conclusions

There is increasing need to evaluate national malaria control programs (and other national public health interventions) using routine data. In this analysis we demonstrate how subnational heterogeneity in ITN coverage can be used to assess a dose–response relationship with HMIS-derived confirmed case incidence, after controlling for important confounding factors. While still an observational study design, the establishment of such a dose–response relationship helps bolster causal inference between ITN program inputs and malaria health outcomes when no true control group is available [38]. Using this approach we provide further evidence that increased coverage with ITNs is associated with decreased malaria morbidity and reduced utilization of health services for malaria illness in Zambia.

## Additional file

Additional file 1:
**Space-time imputation of missing facility-month cases.** **Figure S1**. Bayesian geostatistical estimates of ITN coverage for **(A)** 2008 and **(B)** 2010, Zambia. **Figure S2**. Relative standard deviation (sd to mean ratio) for ITN coverage estimates for **(A)** 2008 and **(B)** 2010, Zambia. **Figure S3**. Population-adjusted ITN coverage estimates by district for **(A)** 2008, **(B)** 2009, **(C)** 2010, and **(D)** 2011, Zambia. **Figure S4**. **(A)** Travel time to the nearest public health facility and **(B)** percent of population by district within 2 hours of a public health facility, Zambia. **Figure S5**. Percent of children <5 with fever in the previous two weeks whose caregiver sought treatment within the public sector, by district, Zambia. **Figure S6**. Map of provinces of Zambia as of 2011, including those defined as high burden. **Figure S7**. District confirmed case incidence (red) and district ITN per HH anomalies (blue), where zero indicates that coverage remained the same as the four-year mean. Months 1-36 refer to the study period January 2009 to December 2011, Zambia. **Table S1**. Results of model selection for models on confirmed cases, 2009-2011 Zambia. **Table S2**. Results of model selection for models on total cases (confirmed + unconfirmed), 2009-2011 Zambia.
