# Supplementary material for: A methodological framework for the improved use of routine health system data to evaluate national malaria control programs: evidence from Zambia
Source: Popul Health Metr. 2014 Nov 19;12:30. doi: 10.1186/s12963-014-0030-0 (PMC4247605; doi:10.1186/s12963-014-0030-0)
Supplement: Additional file 1: — Space-time imputation of missing facility-month cases. Figure S1. Bayesian geostatistical estimates of ITN coverage for (A) 2008 and (B) 2010, Zambia. Figure S2. Relative standard deviation (sd to mean ratio) for ITN coverage estimates for (A) 2008 and (B) 2010, Zambia. Figure S3. Population-adjusted ITN coverage estimates by district for (A) 2008, (B) 2009, (C) 2010, and (D) 2011, Zambia. Figure S4. (A) Travel time to the nearest public health facility and (B) percent of population by district within 2 hours of a public health facility, Zambia. Figure S5. Percent of children <5 with fever in the previous two weeks whose caregiver sought treatment within the public sector, by district, Zambia. Figure S6. Map of provinces of Zambia as of 2011, including those defined as high burden. Figure S7. District confirmed case incidence (red) and district ITN per HH anomalies (blue), where zero indicates that coverage remained the same as the four-year mean. Months 1-36 refer to the study period January 2009 to December 2011, Zambia. Table S1. Results of model selection for models on confirmed cases, 2009-2011 Zambia. Table S2. Results of model selection for models on total cases (confirmed + unconfirmed), 2009-2011 Zambia. [file 12963_2014_30_MOESM1_ESM.docx]

**Additional file 1**

**Space-time imputation of missing facility-month cases**

We adapted a method presented previously by Gething and colleagues (2006) for a dataset with a far greater amount of missing data.^1^ We first created Voronoi tessellations within R^2^ of the HMIS health facilities for which we had latitude and longitude information. A Voronoi tessellation produces polygons from line segments surrounding each point that correspond to all points equidistant from the two nearest sites, and can be used as a rough approximation of a health facility catchment area.^3^ We created separate tessellations for hospitals, health centers, and health posts. Using each tessellation, we created a matrix of neighborhood weights based upon distance, where all facilities within the maximum distance between any two facilities were considered neighbors. We then imputed missing values using conditional autoregressive models (CAR) with existing data values,^4^ the spatial neighboring relationship, a first-order temporal autoregressive term within WinBUGS,^5^ and the estimated facility catchment size. Facility catchment sizes were estimated from the monthly average outpatient attendance for all diagnoses in 2011. For each model, after removing the first 5,000 sampling iterations we sampled every 10^th^ value from the next 10,000 iterations and extracted the median imputed value for the counts at each missing health facility-month cell from the posterior distribution predicted by the model. For the remaining health facilities without global positioning system (GPS) data, we constructed CAR models that incorporated all facilities, including the imputed values from the first model, and additionally included an exchangeable random effect for each facility and a spatial random effect at the district level.

We ran validity checks on this prediction method by withholding a subset of the data for prediction (15%) and training the model on the remaining 85% of the data. We did not identify any systematic bias in predictions by facility type, district, or month. We found a mean prediction error of -3.7 for health centers, -1.6 for hospitals, and -0.81 for health posts. The total mean prediction error was only -3.4, indicating very little bias. Total prediction error was roughly 9%, which is slight considering we imputed 21% of total case values and 37% of confirmed case values.

Additionally, we ran a sensitivity analysis to assess the influence of outliers whereby we ran the initial imputation model with all available data, and ranked the residuals from predictions from this model. We then removed the bottom 2.5% and top 2.5% of these residuals (potentially low and high implausible values based upon the entire dataset). We then reran the regression models without these outliers and obtained similar effect estimates in final models, thereby indicating that potential outliers were not driving our findings.

**ITN coverage estimates**

To produce ITN coverage estimates for each district over the study period the following steps were completed to combine available household survey and enumeration data and program distribution data. In step one, data were first compiled at the cluster level from the 2008 and 2010 MIS surveys, district-level surveys conducted in Luangwa and Nyimba districts, and program household enumeration data on the total number of ITNs and the total number of household members per cluster. There were 400 clusters included for 2008, and 245 clusters for 2010. The ratio of ITNs to persons at each cluster was then modeled in a Bayesian geostatistical framework with a normal (Gaussian) prior and with covariates urban/rural and distance to the district health office. An exponential spatial decay parameter was included to capture spatial autocorrelation between clusters and allow creation of a spatial prediction surface. All models were fit using WinBUGS. Model convergence was assessed using plots of ergodic averages after a burn-in period of 5,000 iterations. For the geostatistical models, predictions to each 5km x 5km grid cell covering Zambia (a total of 36,159 grid cells) were produced using the *spatial.unipred* command (Figures S1 & S2). We conducted a validation exercise whereby we withheld a 15% subset of data for prediction, and ran each model on the remaining 85% training dataset. We found consistent slight under-predictions (mean prediction error of -0.10 in 2008 and -0.05 in 2010) from these validation models, but prediction errors were not spatially patterned. 61% of true values fell within the 95% Bayesian Credible Interval (BCI) in 2008, and 83% of true values fell within the 95% BCI in 2010.

We calculated population-adjusted district means from the predictions of these models by multiplying each ITN per person surface with a Landscan population raster^6^ adjusted for annual population change. In step two, we used program ITN distribution data and district populations for each year to separately predict the potential availability of ITNs per person per district from distributions over the study period by applying a decay function based upon the NetCalc algorithm to the previous three years’ distribution counts.^7,8^ This algorithm assumed a three-year half-life for ITN decay. Finally, in step three we built a linear regression model with random intercepts for each district to assess the relationship between the predicted availability from distribution data and the modeled coverage estimates for 2008 and 2010 from step one, and used this model to interpolate ITN per person estimates for 2009 and 2011 (Figure S3). Model assessment revealed that incorporation of spatial effects in step three did not improve model fit and therefore only uncorrelated random intercepts were included in this final interpolation step.

**Figure S1. Bayesian geostatistical estimates of ITN coverage for (A) 2008 and (B) 2010, Zambia.**

**A. 2008 mean ITNs:Person** **B. 2010 mean ITNs:Person**


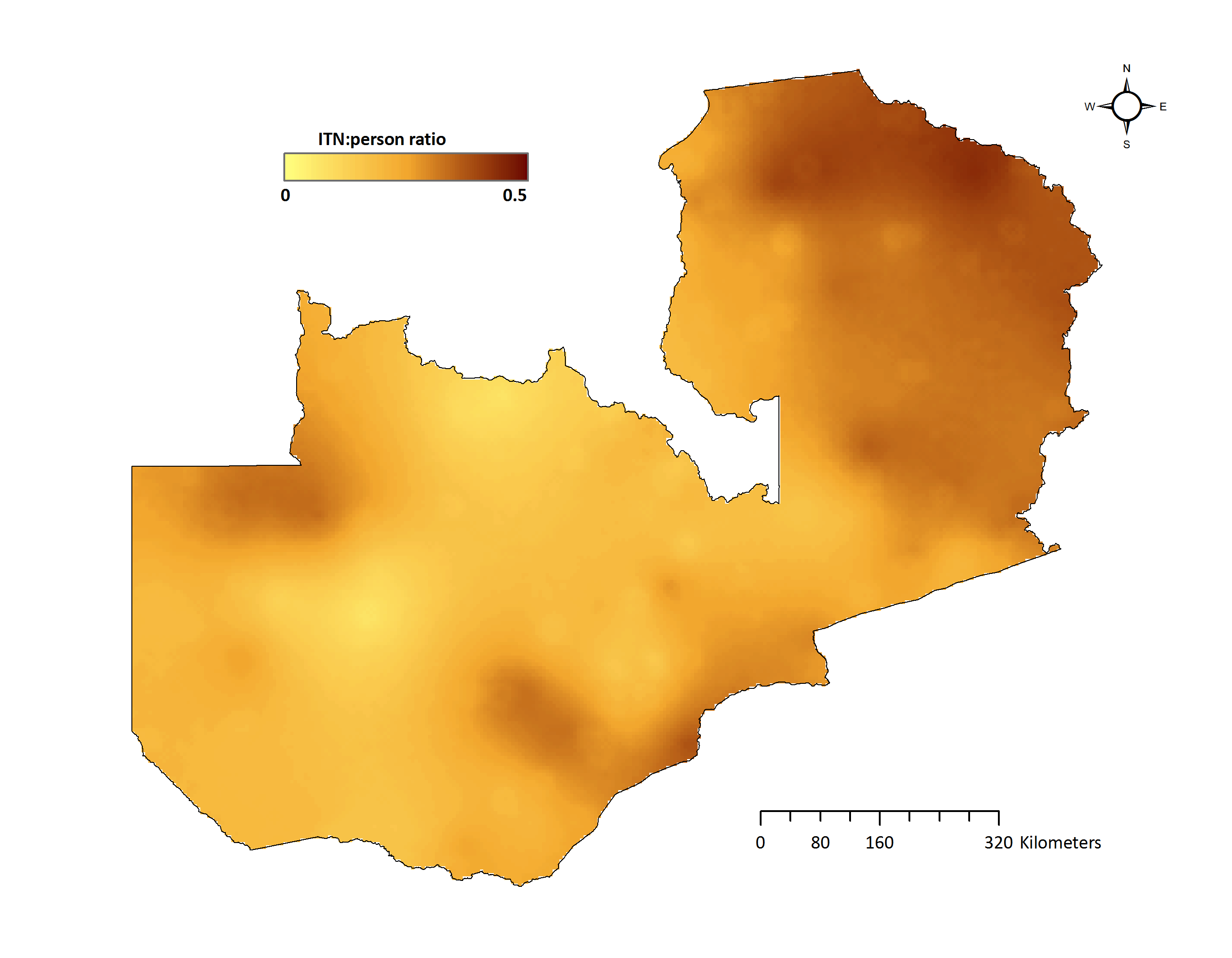

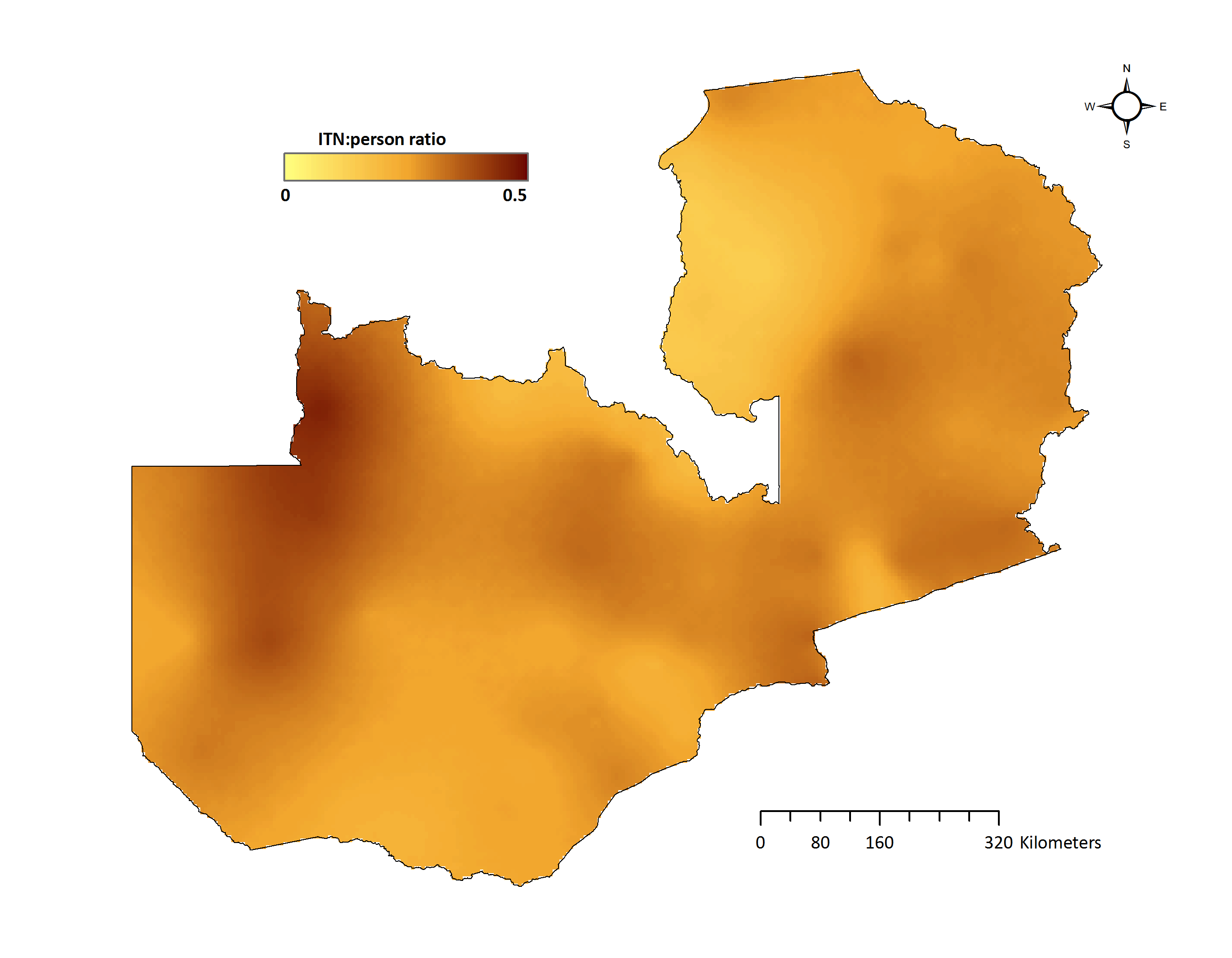


**Figure S2. Relative standard deviation (sd to mean ratio) for ITN coverage estimates for (A) 2008 and (B) 2010, Zambia.**

**A. 2008 Standard deviation to mean ratio B. 2010 Standard deviation to mean ratio**


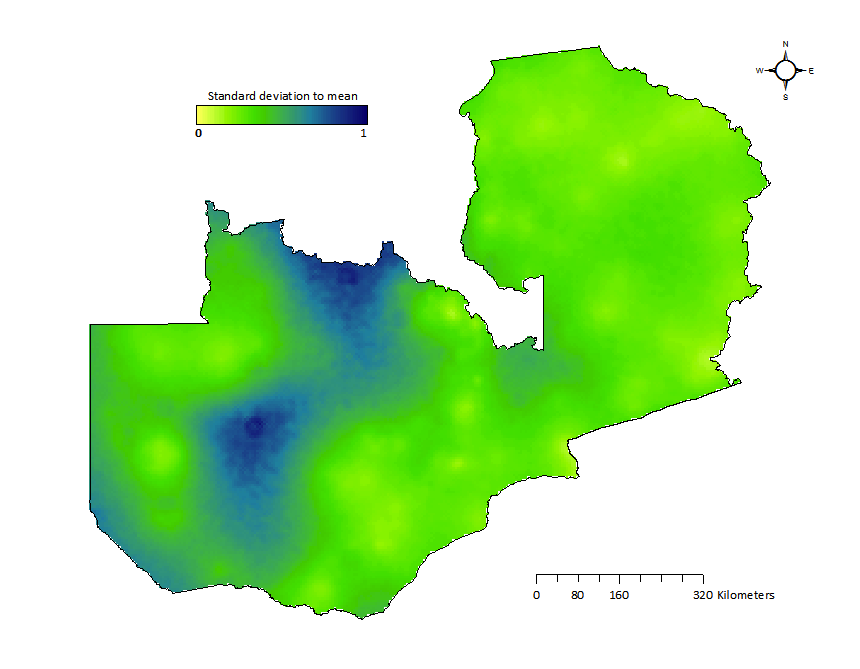

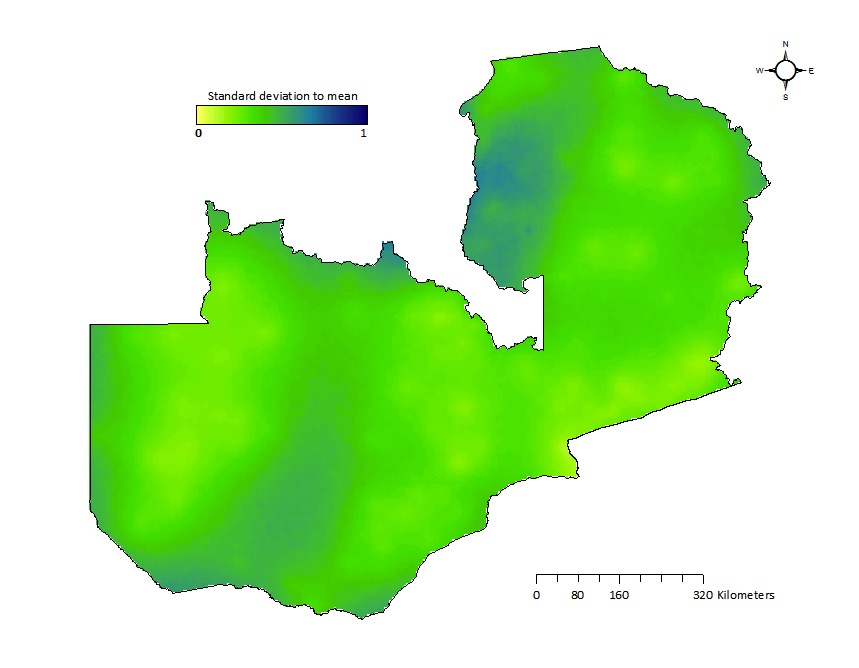


**Figure S3. Population-adjusted ITN coverage estimates by district for (A) 2008, (B) 2009, (C) 2010, and (D) 2011, Zambia.**

**A. 2008 ITNs:Person by district**  **B. 2009 ITNs:Person by district**


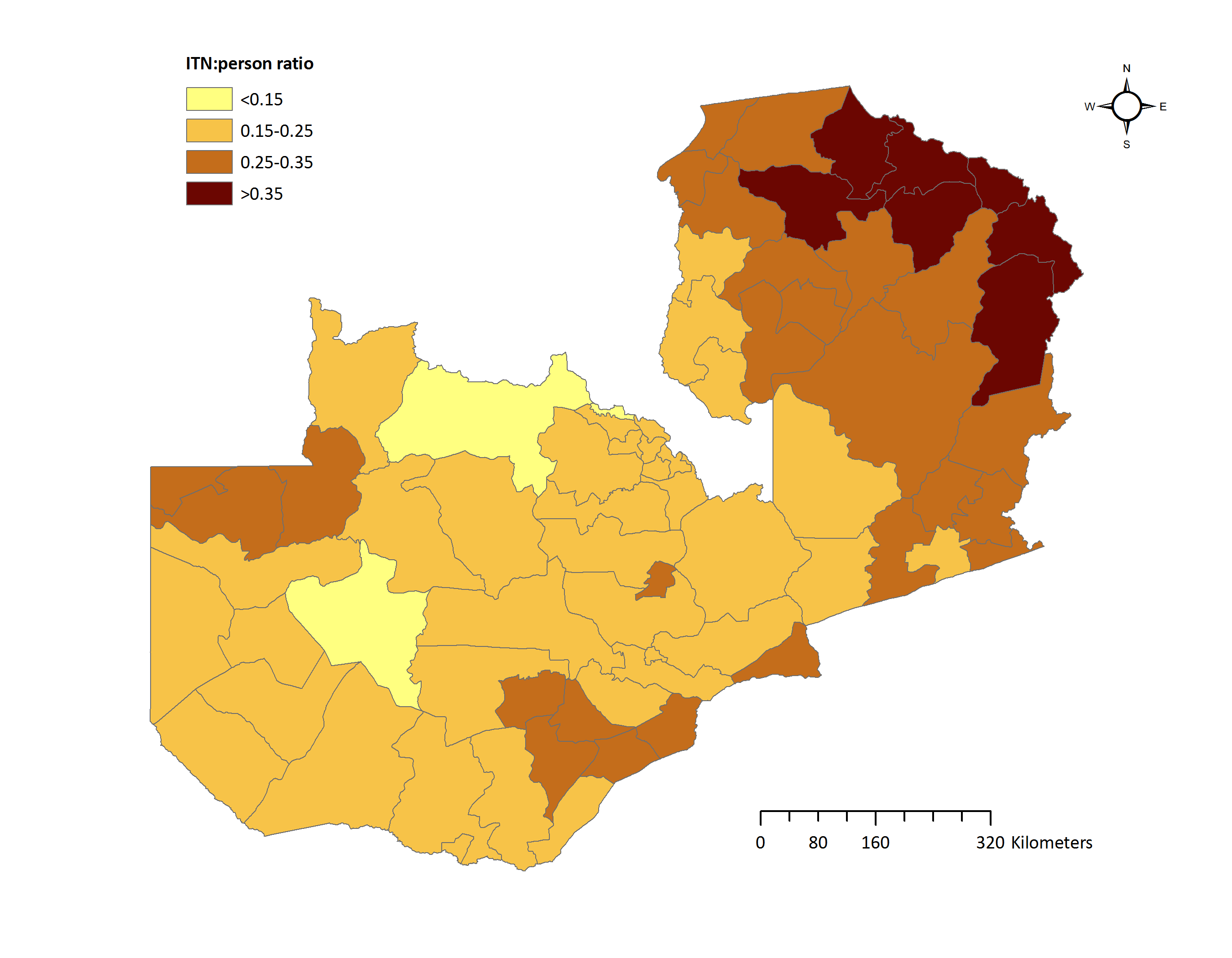

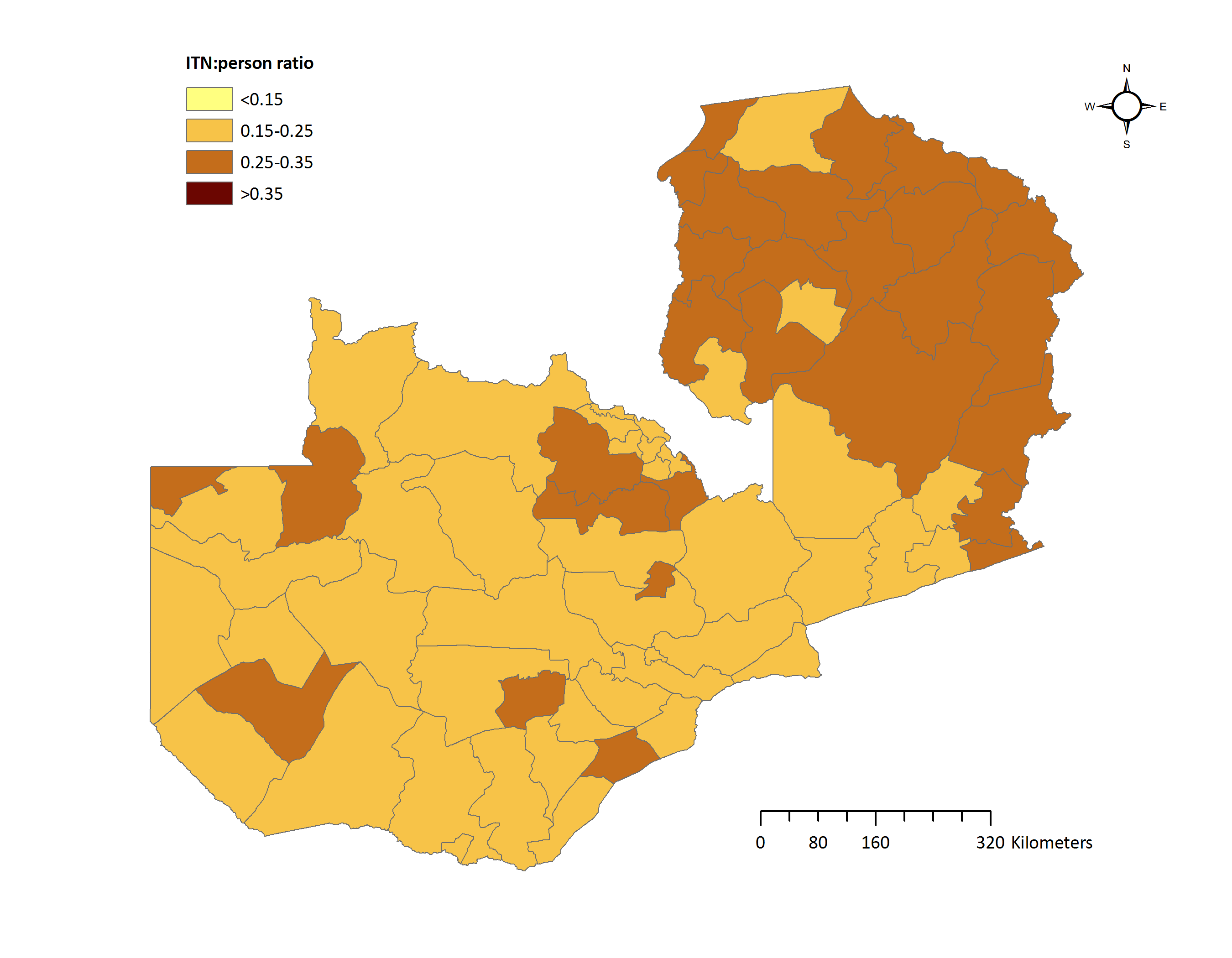


**C. 2010 ITNs:Person by district**  **D. 2011 ITNs:Person by district**


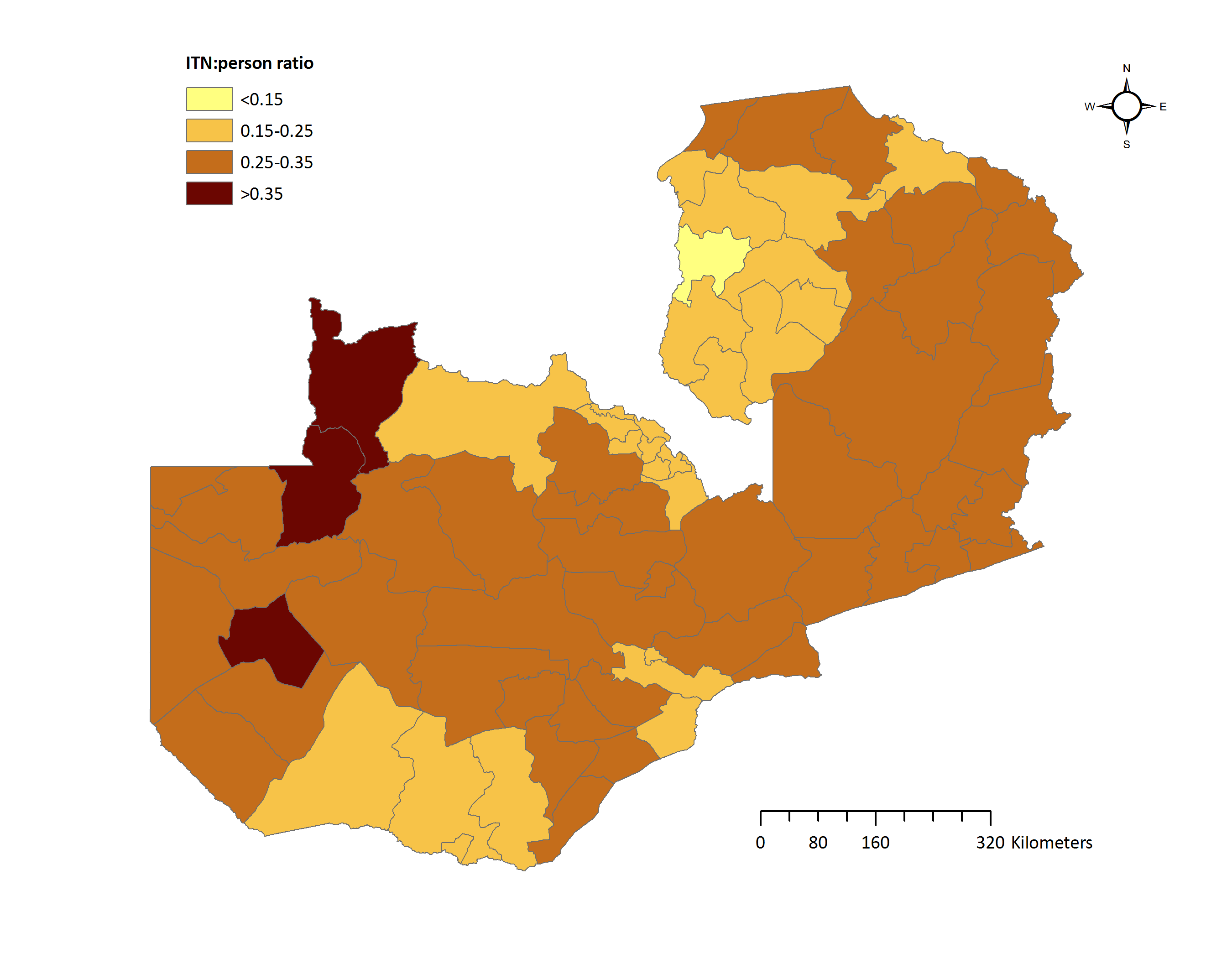

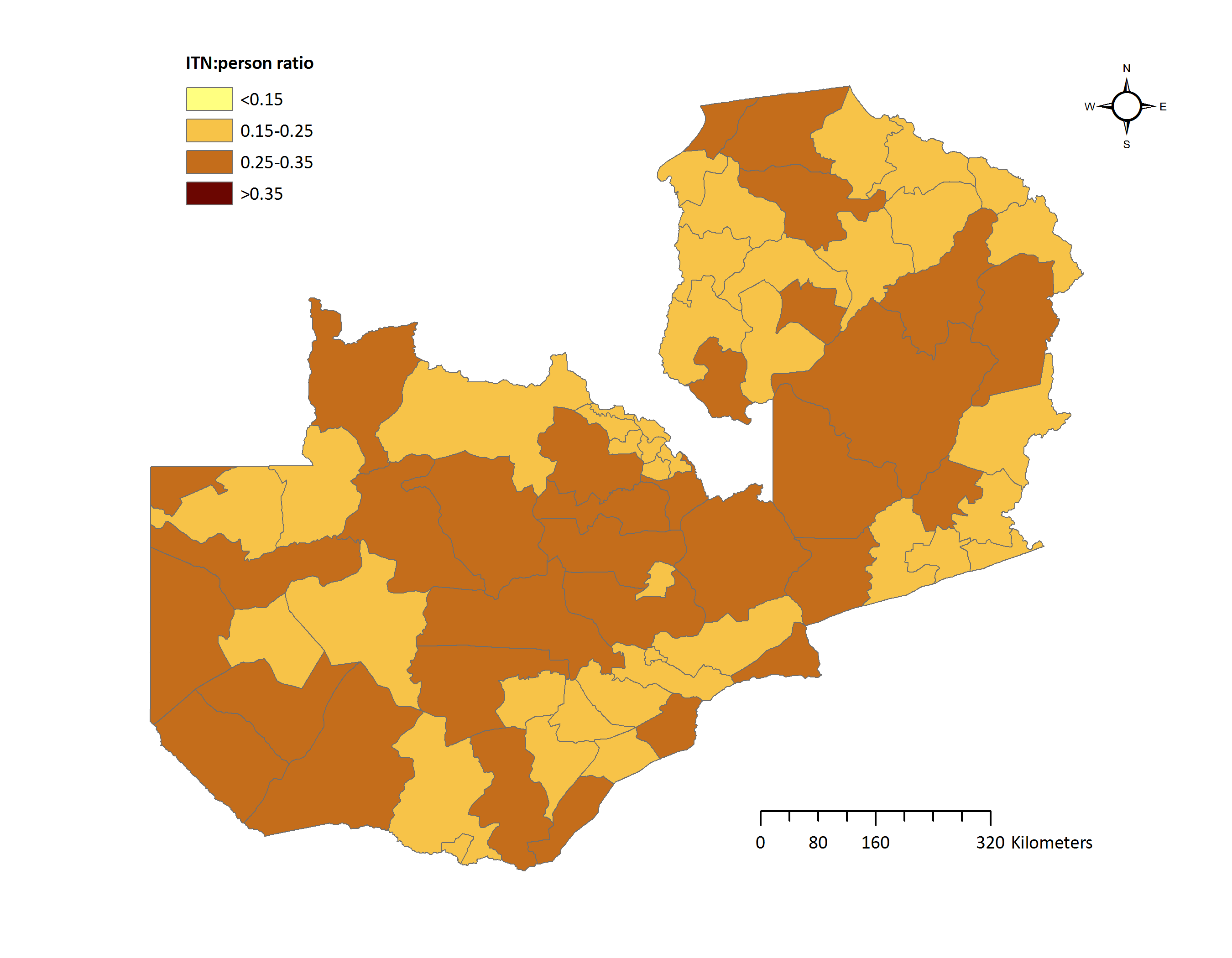


**Health facility access**

To produce the AccessMOD travel time surface, geographic layers were incorporated for land use from Globcover,^9^ for altitude from USGS,^10^ for population from Landscan 2008,^6^ and for health facilities and road networks from administrative shape files. AccessMOD produces an estimated travel time per raster cell based on assumed travel speeds over various land use types, roads, and altitude gradients. This raster was overlaid with the Landscan population raster to estimate for each district the proportion of the population within 2 hours of a health facility (Figure S4).

**Figure S4. (A) Travel time to the nearest public health facility and (B) percent of population by district within 2 hours of a public health facility, Zambia.**


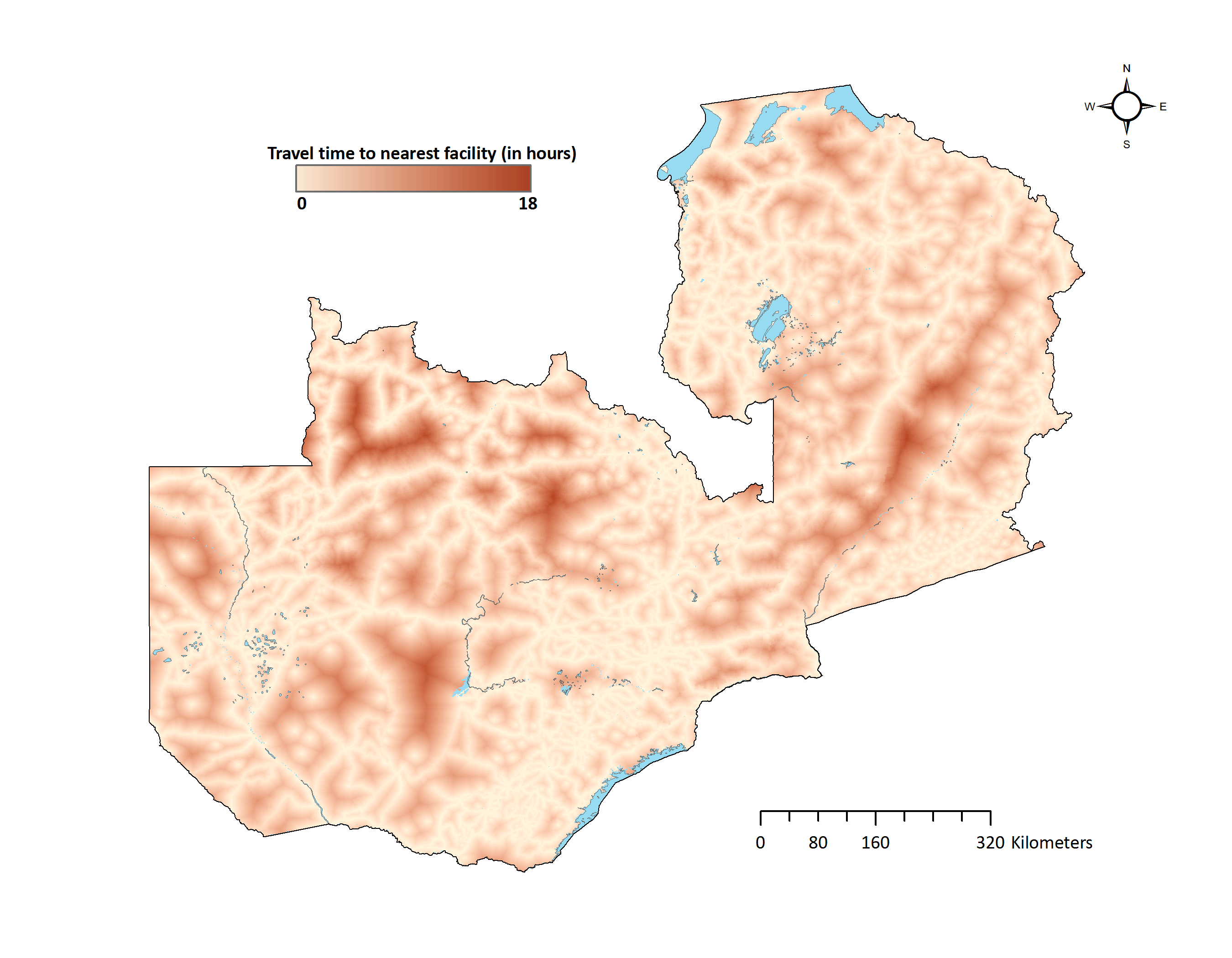

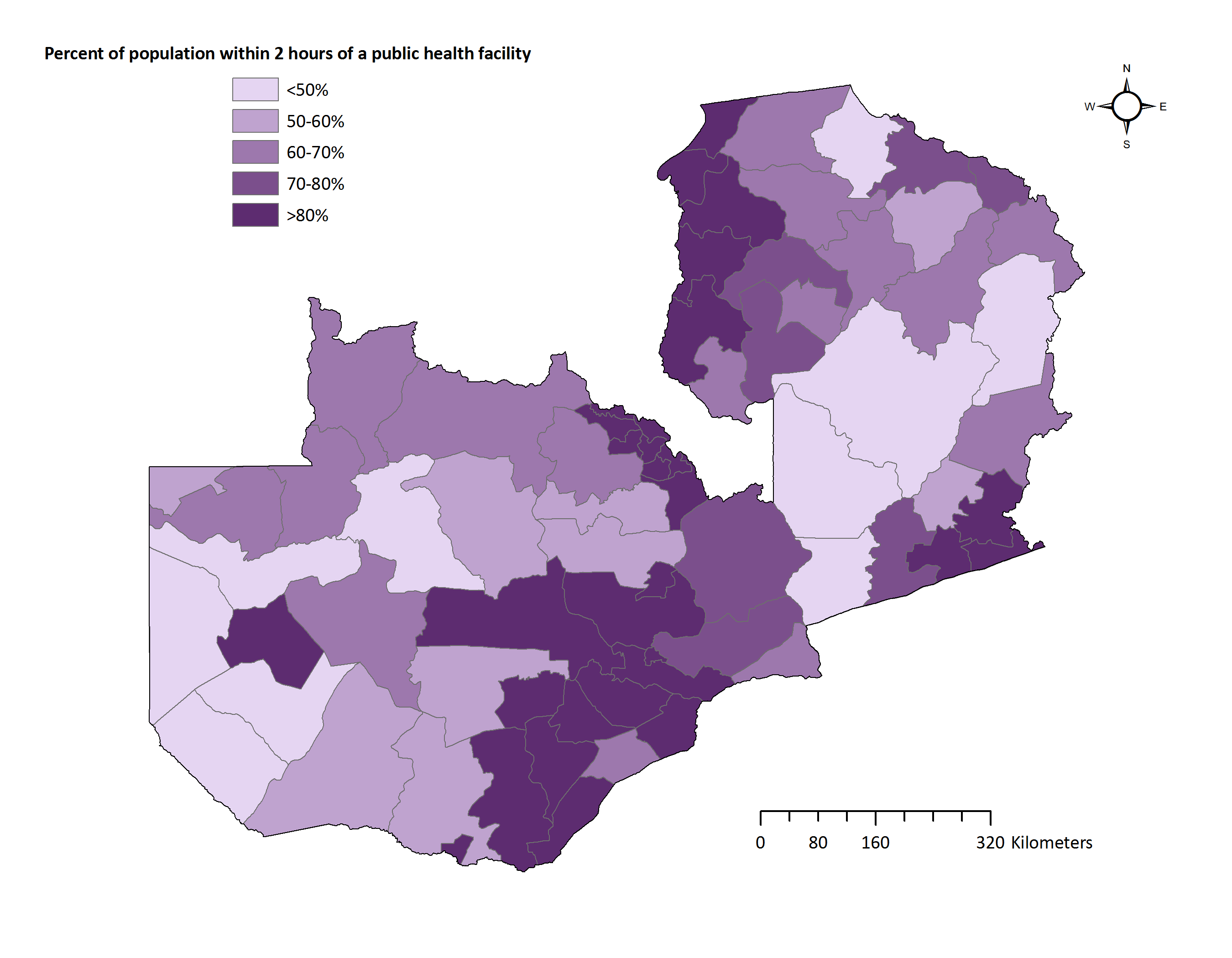


**Treatment-seeking rates**

**Figure S5. Percent of children <5 with fever in the previous two weeks whose caregiver sought treatment within the public sector, by district, Zambia.**


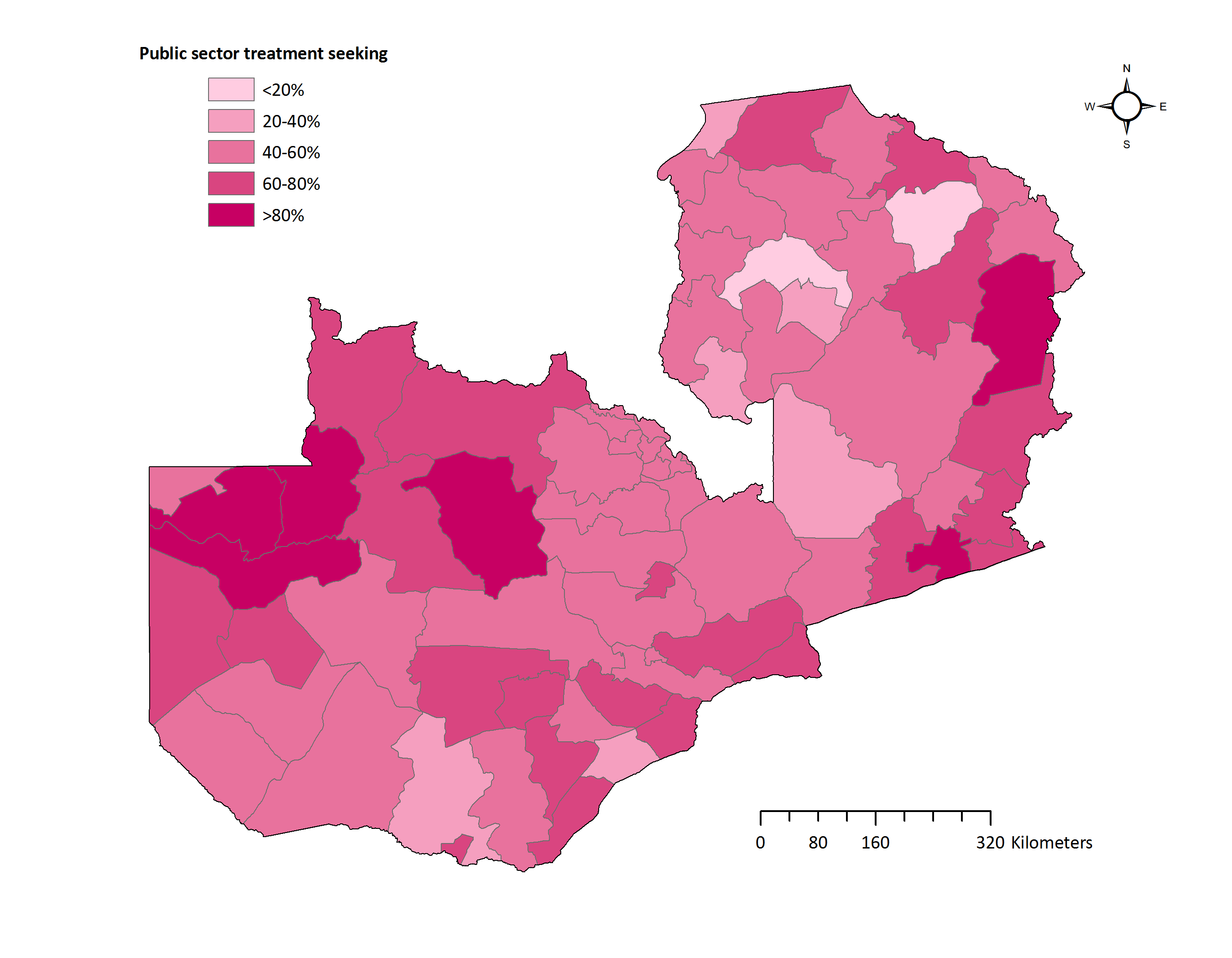


**Figure S6. Map of provinces of Zambia as of 2011, including those defined as high burden.**


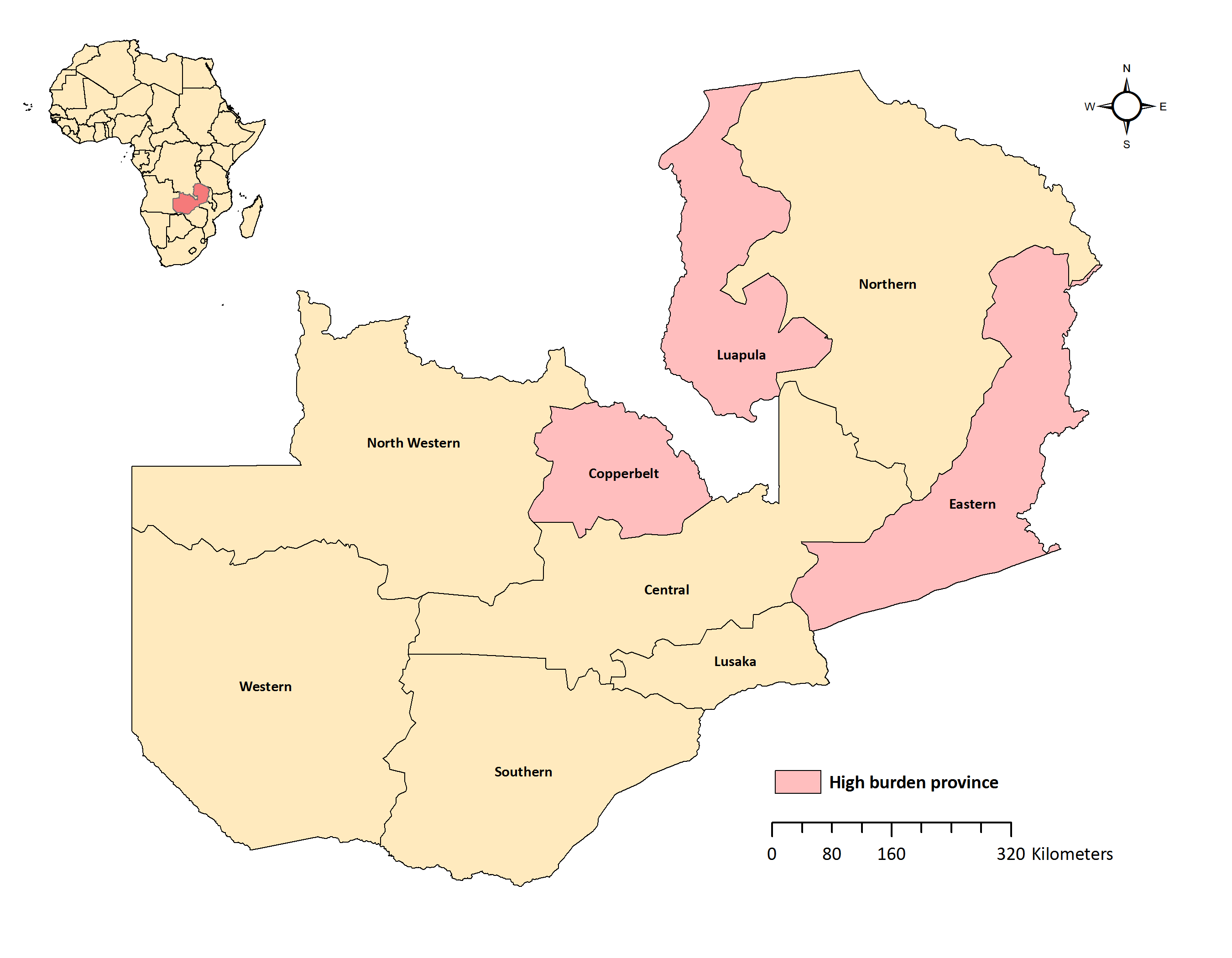


**Descriptive results by district**

**Figure S7. District confirmed case incidence (red) and district ITN per HH anomalies (blue), where zero indicates that coverage remained the same as the four-year mean. Months 1-36 refer to the study period January 2009 to December 2011, Zambia.**

**Model selection with INLA**

The best-fitting final models according to the deviance information criterion (DIC) included the interaction between ITN coverage and high burden province, fixed effects for district reporting and testing rates, treatment seeking, health care access, maximum and minimum temperature, rainfall, vegetation index, calendar month, year, and both uncorrelated and spatially correlated random effects for district (Tables S1 & S2).

**Table S1. Results of model selection for models on confirmed cases, 2009-2011 Zambia.**

| **Model specification** | **DIC** | **ITN coefficient [IRR (95% BCI)]** |
| --- | --- | --- |
| 1. Fixed district covariates + reporting + testing + year + month | 38333.4 |  |
| 2. Model 1 + climate | 38301.1 |  |
| 3. Model 2 + ITNs per HH | 38262.7 | 0.71 (0.63 – 0.79) |
| 4. Model 3 + IRS | 38241.0 | 0.73 (0.65 – 0.81) |
| 5. Model 4 non-spatial | 38241.2 | 0.74 (0.66 – 0.83) |
| 6. Model 4 with ITNs:region interaction | 38225.8 | Low: 0.59 (0.51 – 0.68)  High: 0.94 (0.79 – 1.10) |

**Table S2. Results of model selection for models on total cases (confirmed + unconfirmed), 2009-2011 Zambia.**

| **Model specification** | **DIC** | **ITN coefficient [IRR (95% BCI)]** |
| --- | --- | --- |
| 1. Fixed district covariates + reporting + testing + year + month | 42992.1 |  |
| 2. Model 1 + climate | 42948.9 |  |
| 3. Model 2 + ITNs per HH | 42900.5 | 0.68 (0.61 – 0.76) |
| 4. Model 3 + IRS | 42901.9 | 0.69 (0.62 – 0.76) |
| 5. Model 4 non-spatial | 42902.7 | 0.68 (0.61 – 0.76) |
| 6. Model 4 with ITNs:region interaction | 42878.1 | Low: 0.53 (0.46 – 0.62)  High: 0.93 (0.81 – 1.10) |

**References**

1. Gething PW, Noor AM, Gikandi PW, Ogara EA, Hay SI, Nixon MS, et al: **Improving imperfect data from health management information systems in Africa using space-time geostatistics.** *Plos Med* 2006; **3**(6): e271.

2. R Computing Team: **R: A language and environment for statistical computing.** *R Foundation for Statistical Computing* 2013; Vienna, Austria.

3. Gething PW, Noor AM, Goodman CA, et al: **Empirical modelling of government health service use by children with fevers in Kenya.** *Acta Trop* 2004; **91**(3): 227-37.

4. Clayton DG, Bernardinelli L, Montomoli C: **Spatial correlation in ecological analysis*.*** *Int J Epidemiol* 1993; **22**(6): 1193-202.

5. Lunn DJ, Best TA, Spiegelhalter D: **WinBUGS--a Bayesian modelling framework: concepts, stucture, and extensibility.** *Statistics and Computing* 2000; **10**: 325--337.

6. UT-Battelle. Landscan 2008. [accessed 2011 January]; Accessed online at: <http://www.ornl.gov/sci/landscan/>.

7. Briet OJ, Hardy D, Smith TA: **Importance of factors determining the effective lifetime of a mass, long-lasting, insecticidal net distribution: a sensitivity analysis.** *Malar J* 2012; **11**: 20.

8. Yukich J, Bennett A, Keating J, Yukich RK, Lynch M, Eisele TP, et al: **Planning long lasting insecticide treated net campaigns: should households' existing nets be taken into account?** *Parasit Vectors* 2013; **6**: 174.

9. ESA. GlobCover 2.3. 2009 [accessed 2012 February]; Accessed online at: <http://ionia1.esrin.esa.int/>.

10. United States Geological Survey. Hydro 1k. [accessed 2011 January]; Accessed online at: <http://eros.usgs.gov/#/Find_Data/Products_and_Data_Available/HYDRO1K>.
